# Central Pattern Generator (CPG)-Based Locomotion Control and Hydrodynamic Experiments of Synergistical Interaction between Pectoral Fins and Caudal Fin for Boxfish-like Robot

**DOI:** 10.3390/biomimetics8040380

**Published:** 2023-08-21

**Authors:** Lin Chen, Yueri Cai, Shusheng Bi

**Affiliations:** Robotics Institute, School of Mechanical Engineering and Automation, Beihang University, Beijing 100191, China; by1907004@buaa.edu.cn (L.C.); caiyueri@buaa.edu.cn (Y.C.)

**Keywords:** robotic fish, CPG, motion control, multifin synergy, hydrodynamic analysis

## Abstract

Locomotion control of synergistical interaction between fins has been one of the key problems in the field of robotic fish research owing to its contribution to improving and enhancing swimming performance. In this paper, the coordinated locomotion control of the boxfish-like robot with pectoral and caudal fins is studied, and the effects of different control parameters on the propulsion performance are quantitatively analyzed by using hydrodynamic experiments. First, an untethered boxfish-like robot with two pectoral fins and one caudal fin was designed. Second, a central pattern generator (CPG)-based controller is used to coordinate the motions of the pectoral and caudal fins to realize the bionic locomotion of the boxfish-like robot. Finally, extensive hydrodynamic experiments are conducted to explore the effects of different CPG parameters on the propulsion performance under the synergistic interaction of pectoral and caudal fins. Results show that the amplitude and frequency significantly affect the propulsion performance, and the propulsion ability is the best when the frequency is 1 Hz. Different phase lags and offset angles between twisting and flapping of the pectoral fin can generate positive and reverse forces, which realize the forward, backward, and pitching swimming by adjusting these parameters. This paper reveals for the first time the effects of different CPG parameters on the propulsion performance in the case of the synergistic interaction between the pectoral fins and the caudal fin using hydrodynamic experimental methods, which sheds light on the optimization of the design and control parameters of the robotic fish.

## 1. Introduction

As the earliest vertebrates on the earth, with millions of years’ evolution, fish have shown amazing swimming performance, with extraordinary swimming speed, excellent maneuverability, superior stability, and efficient energy utilization [1]. Inspired by biological systems, studying the swimming morphology and swimming behavior of fish provides an important reference for the design and optimization of bionic robotic fish [2,3,4]. Since the advent of the first robotic fish RoboTuna [5], scholars have used various technical means to analyze the structural characteristics and swimming patterns of biological fish to achieve efficient swimming and improve the swimming performance of robotic fish. The commonly used analytical methods to guide the design of robotic fish include kinematics research [6], computational fluid dynamics (CFD) [7], intelligent control algorithm research [8], etc. Moreover, smart materials have also been applied to the component design of robotic fish, such as the use of ion polymer metal composites (IPMC), shape memory alloy (SMA), and piezoelectric materials to design the actuation system of robotic fish [9,10,11]. With the development of communication and sensing technologies, developing efficient, flexible, and stable robotic fish for underwater operations in complex environments has become a goal pursued by researchers [12,13]. As an important tool for the exploration and development of the ocean, the robot fish overcomes the shortcomings of underwater robots using traditional propellers. It is especially suitable for practical application scenarios such as environmental monitoring, underwater exploration, and marine fishing. The related research is attracting increasingly more attention from scholars [14].

Breder [15] proposed the classification scheme and nomenclature of fish according to the type of swimming. Lindsey [16] and Webb [17] concluded the above classification into two propulsive modes according to the body parts that generate propulsion when swimming: body and/or caudal fin propulsion (BCF) mode, and media and/or paired fin propulsion (MPF) mode [18]. The swing of the caudal fin can generate a backward propulsion wave, which is conducive to the continuous and fast swimming of fish. Therefore, 85% of fish use the BCF mode for propulsion. Most fishes use dorsal, pectoral, pelvic, and anal fins for assisted propulsion and posture adjustment. The MPF-mode fish use these fins as the main propulsion components, which have better stability and maneuverability [18]. However, these two movement modes are not completely independent, and most fishes combine the two modes of swimming—using the high propulsion generated by the swing of the caudal fin to swim at high speed and using the pectoral, dorsal, and anal fins to adjust the movement posture and maintain some directional stability. For example, the boxfish (ostraciiform) has a relatively rigid body and caudal fin. Although their hydrodynamic efficiency is not as good as thunniform swimmers, they can achieve high-speed movement by spreading out their caudal fin and swinging like a pendulum [19]. The box-like body can achieve good maneuverability and maintain the stability of its posture by retracting the caudal fin and through the coordination of the pectoral, dorsal, and anal fins. Therefore, researchers have conducted in-depth exploration and research on how the coupled cooperative relationship between multiple fins affects the swimming performance of fish.

The mutual coordination among multiple fins in fish plays a crucial role in determining the propulsion performance, stability, and maneuverability of robotic fish. Consequently, the investigation of the interaction mechanism between fins in fish and its application in guiding the design and enhancement of robotic fish, with the aim of improving swimming performance, has garnered considerable interest among scholars in the field. Mignano et al. [20] developed a bionic robot platform with multiple fins and conducted a comprehensive study on the impact of the relative phase and position of various fins, such as the caudal peduncle and dorsal fin, anal fin, and caudal fin, on the thrust generated by the prototype. Through a combination of experimental device measurements and two-dimensional CFD methods, they were able to determine the optimal phase relationship. The results revealed that selecting the appropriate phase relationship can provide more than double the average thrust and a substantial reduction in lateral force. Matthews et al. [21] designed a fish-like robot model and studied the effects of the distance and relative movement between middle fins arranged in series on the motion pattern of fish. The results revealed that when the middle fins of undulatory fishes move at high frequency, the movement between fins which are out of phase will reduce swimming speed by 12–26%. Optimizing the positioning and relative movement of multiple fins is crucial for minimizing oscillations during swimming and enhancing overall performance. While researchers have conducted simulation and experimental studies on the swing position and phase relationship of various fins, the majority of these investigations have primarily focused on analyzing the interaction between the dorsal fin, anal fin, and caudal fin. However, there is a scarcity of research on the effects of the synergistic interaction between the pectoral fins and caudal fin on the propulsion performance of robotic fish. Zhang et al. [22] designed a robotic fish equipped with symmetrical bionic pectoral long fins and double-jointed caudal fins. By utilizing a combination of mixed synergistic propulsion from the pectoral and caudal fins, the robotic fish demonstrated exceptional stability and propulsion performance during low-speed swimming. This study provides evidence that the hybrid propulsion of pectoral and caudal fins enhances the movement maneuverability of robotic fish, showcasing its potential for improved swimming capabilities. Sharifzadeh et al. [23] designed a fish-inspired robot with two degree-of-freedom pectoral fins and a single degree-of-freedom caudal fin and used a CMA-ES-assisted workflow to train goal-specific swimming gaits. The results show that the robotic fish can reach a forward swimming speed of 0.385 m/s (0.71 body lengths per second) and achieve a near-zero turning radius. Drago et al. [24] used reinforcement learning method to determine CPG controllers that produce propulsively beneficial kinematics in a multifin underwater robot. Due to the fact that the pectoral and caudal fins are the primary propulsion components of biological fish, many scholars have studied the effects of different forms of pectoral and caudal fins on swimming, including rigid, multibody and soft fins [25,26,27]. Oiu et al. [28] reveals for the effect of synergistic interactions between pectoral and caudal fins on the stability of body’s course by means of Computational Fluid Dynamics and prototype experiments. They analyzed the influence of the pectoral and caudal fin synergistically interactions on the heading stability of the prototype and proposed that proper coordination parameters of pectoral and caudal fins can improve the heading stability of robotic fish. At the same time, appropriate synergistic parameters can also improve the locomotion performance and swimming efficiency of robotic fish. Therefore, a systematic analysis of the propulsion performance of the robotic fish, considering the synergistic interaction between the pectoral fins and caudal fin, is necessary. Additionally, exploring the effects of different CPG control parameters on the propulsion performance of both the pectoral fin and caudal fin will provide valuable insights into the motion characteristics of multiple fins. Moreover, this research will contribute to the enhancement of swimming control algorithms for robotic fish. Furthermore, it is beneficial to improve the swimming performance of robotic fish and provide a guidance for the research of fish movement mechanisms under the synergistic interaction of the pectoral fin and caudal fin.

Motivated by previous research and the work of our team in the field of boxfish-like robots [28], this paper improves the structure, hardware, and control strategy of robotic fish. Based on the designed six degrees of freedom (DOF) fins (double two-DOF pectoral fins and single two-DOF caudal fin), a CPG-based controller is designed. To analyze the impact of the time asymmetric flapping characteristics of the pectoral fin on the propulsion performance of the robotic fish, a frequency continuity transition equation is introduced, allowing for different flapping times of the pectoral fins. To evaluate the propulsion performance of the prototype, a three-axis force experimental platform is constructed to measure the force exerted on the robot under different CPG parameters. Initially, the thrust and lateral force generated by the two-DOF tail joints alone are measured to determine the optimal control parameters for the caudal fin, enhancing the robot fish’s propulsion performance. Subsequently, the hydrodynamic performance under the influence of independent pectoral fins is analyzed to select suitable control parameters. Finally, the average propulsion force of the robotic fish is measured under different CPG parameters, considering the synergistic interaction between the pectoral fins and caudal fin. The effects of the CPG parameters on the propulsion performance of the prototype are analyzed, providing a foundation and experimental guidance for the design of robotic fish and the enhancement of swimming performance. The hydrodynamic performance of the prototype under different CPG parameters is assessed using an underwater experimental device, and the impact of control parameters on propulsion performance in the context of the synergistic interaction between pectoral fins and the caudal fin is investigated. This research offers valuable insights for the design of robotic fish and the improvement of their swimming capabilities.

The rest of the paper is organized as follows: Section 2 presents the structural design of the robotic fish prototype. Section 3 introduces the control system of the robotic fish, including the hardware composition and the control algorithm based on CPG. The hydrodynamic experiments and analysis are carried out on the propulsion performance of the pectoral fins and caudal fin of the prototype with different CPG parameters in Section 4. Conclusions and future work are shown in Section 5.

## 2. Prototype Design

Three main aspects are normally considered in the design of the robotic fish: actuation system, material selection, and structural layout. As the core part of the prototype design, the actuation system aims to realize the swing of the pectoral and caudal fins of the robotic fish to achieve a better swimming effect. In addition, it is necessary not only to obtain high propulsion efficiency and good maneuverability, but also to consider the characteristics of small size and good maneuverability. Material selection needs to consider the swimming environment of the robotic fish while meeting the strength requirements of the actuation system. The structural layout is mainly composed of two parts: shape characteristics and internal space. The shape mainly imitates the functional structure of the biological fish, and the internal space is determined comprehensively according to the hardware structure and control system. In this part, the design of the prototype is divided into two parts: the structural design and the control system. The structural part imitates the shape characteristics and actuation mode of the boxfish-like robot. The control system is used to guide the decision-making execution and information exchange of the prototype to achieve different swimming modes.

**Figure 1:**
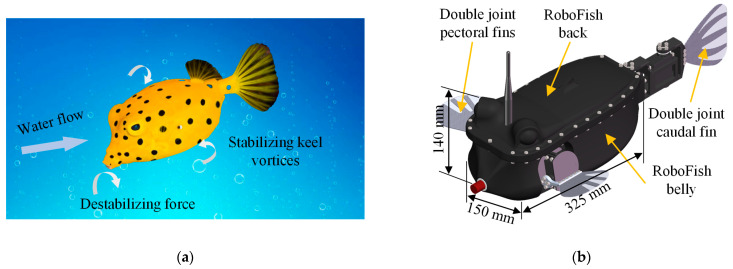
Shape characteristics and actuation mechanism of the bionic boxfish-like robot. (**a**) Swimming instability and maneuverability of boxfish [30]; (**b**) Designed virtual prototype of the boxfish-like robot.

To design a highly maneuverable boxfish-like robot prototype with autonomous swimming capabilities, it is necessary to combine the requirements of mechanical structure, functional characteristics, and perceptual capabilities, so that the prototype has as many equivalent functions as possible compared to its natural biological counterpart while remaining a compact mechanical structure [29]. In other words, biomimetic techniques are utilized to simulate the behavioral characteristics of natural boxfish and to guide the design of the prototype. The exoskeleton of the boxfish consists of a carapace shaped like a box, wrapped by hard scales, and the edge of the carapace is called a keel. The boxfish dynamically adjusts the propulsion speed and posture of the body during swimming through the pectoral fin, caudal fin, dorsal fin, and anal fin. Because of its quadrilateral shell shape, the boxfish creates vortices—keel vortices—around the edge of its body when it swims in the water [30]. Van Wassenbergh et al. [31] showed that the carapace in front of the boxfish creates an unstable force in the boxy front of the boxfish carapace when it swims, thus counteracting the steady state provided by the boxfish keel vortex and making the movement of the boxfish unstable, a characteristic that ensures the motor mobility of the boxfish in the coral reef. Figure 1a illustrates the destabilizing moment caused by the heading attitude change and the stabilizing moment caused by the keel-induced vortex during the swimming of boxfish. Therefore, in addition to relying on the self-stabilizing moment generated by the keel vortex during the swimming process, the boxfish also needs to actively adjust the coordinated motion of coupled multiple fins to maintain the stability of the heading and attitude. Furthermore, boxfish also need to actively control the synergy of multiple fins to achieve smooth switching of multimodal movements (such as turning, floating up, diving, etc.). That is, the boxfish realizes the dynamic unity of maneuverability and stability through coupled interaction between multiple fins [32].

Considering the compactness of the structure and reducing the complexity of the control algorithm, the dorsal and anal fins of the natural boxfish were omitted in the design of the robotic fish. On the one hand, compared with the pectoral and caudal fins, the dorsal and anal fins play a smaller role; on the other hand, at this stage, we mainly study the propulsion performance under the synergistic interaction of pectoral and caudal fins. The virtual prototype of the robotic fish is designed according to the shape characteristics and swimming patterns of the biological boxfish, as shown in Figure 1b. Both the pectoral fins and caudal fin of the robotic fish are driven by servomotors. The pectoral fins on both sides of the prototype have two degrees of freedom (DOFs) of twisting and flapping, which can simulate the movements of boxfish in lift-based swimming mode and drag-based swimming mode [33,34,35]. The tail of the prototype adopts two servomotors for double-joint serial connection, which enhances the motion transmission capability of the caudal fin and is conducive to the stable swing of the caudal fin. The main structure of the prototype is assembled from two parts, the back and the abdomen, to form a sealed cabin, which is made of a photosensitive resin material named DSMIMAGE8000 using 3D printing technology. The length, width, and height of the main body of the designed prototype are 325 mm, 150 mm, and 140 mm, respectively.

**Figure 2:**
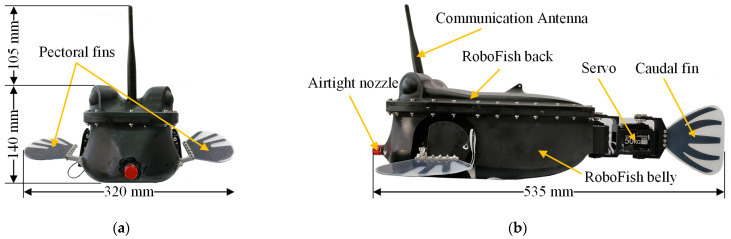
Actual prototype of boxfish-like robot. (**a**) Front view; (**b**) Side view.

The physical object and overall three-dimensional (3D) size of the designed robotic fish are shown in Figure 2. Based on our previous work [28], the pectoral and caudal fins of the prototype are made of carbon fiber plates (CFP) and silicone. Meanwhile, part of the carbon fiber plate skeleton of the fin is hollowed out along the radial direction and filled with silicone glue, which endows the fin surface with flexibility and improves the bionic performance of the fin surface flapping. The simulation result using one/two-way fluid-structure interaction (FSI) numerical analysis method shows that [28]: 0.2 mm carbon fiber skeleton for pectoral fins and 0.3 mm carbon fiber skeleton for caudal fin help improve the propulsion performance of the prototype while swimming.

**Figure 3:**
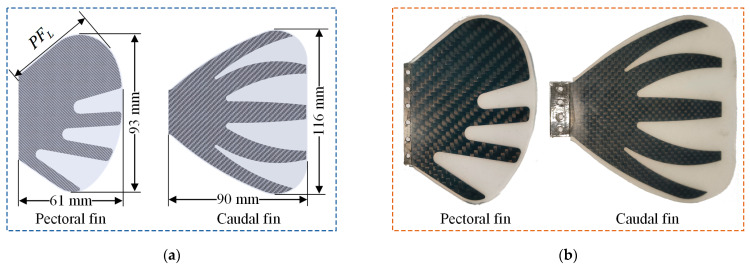
Designed and actual illustration of the pectoral fin and caudal fin of the prototype. (**a**) Shape characteristic parameters of the pectoral and caudal fins; (**b**) Shape characteristics of the actual pectoral and caudal fin.

Figure 3 shows the shape characteristics and dimensions of the pectoral and caudal fins of the designed prototype. Some scholars have studied the relationship between the shape of the pectoral fins and the swimming velocity of fish swimming with median and/or paired fin (MPF) mode and found that the fishes which swim at a slow speed have more symmetrical circular pectoral fins [36,37]. In addition, the shape of the fin has no obvious correlation with body size. Many variables describe the differentiation of fin shape, one of which is the aspect ratio (*A_r_*), that is, a measure of fin length relative to the width. The aspect ratio of the pectoral fin is calculated as follows:

(1) $$A_{r}=\frac{PF_{L}^{2}}{S}$$

where $PF_{L}$ is the length of the pectoral fin anterior margin, and S is the surface area of the pectoral fin. For fishes propelled by pectoral fins (such as labriform), the shape of the pectoral fin ranges from a low aspect ratio of paddle fins (i.e., *A_r_* ≤ 2.0) to a relatively high aspect ratio (i.e., *A_r_* ≥ 4.5) [37]. Naser et al. [38] studied the effects of different shapes of pectoral fins on swimming velocity and efficiency and showed that high aspect ratio pectoral fins have less propulsion resistance and high propulsion efficiency, but they are more likely to lead to heading deviation and affect stability. Considering the stability and maneuverability of the prototype during swimming, the shape characteristics of the designed pectoral fin are shown in Figure 3, and its aspect ratio is 0.71.

Both the pectoral fins and the caudal fins have two DOFs and are controlled by two servomotors, respectively. To imitate the swing characteristics of biological fish fins, the rotation angle of the servomotor is mechanically limited to avoid interference between motion joints. Table 1 shows the characteristic parameters of the pectoral and caudal fins.

## 3. Control System

### 3.1. Hardware Structure

**Figure 4:**
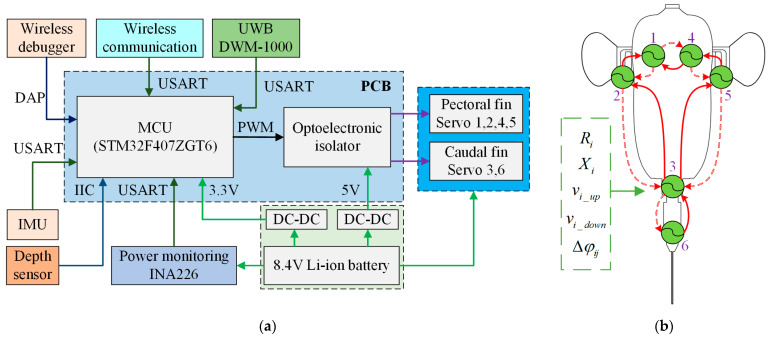
Control system of designed boxfish-like robot prototype. (**a**) Hardware system structure; (**b**) Designed artificial CPG network.

The hardware structure of the prototype is mainly composed of a power supply module, a communication module, sensors, and a program downloader, as shown in Figure 4a. The power supply module consists of a lithium battery (with a capacity of 1000 mAh and a rated output voltage of 8.4 V), a power detection module and a DC-DC module (for voltage conversion). The power monitoring module is used to monitor the status of the battery in real-time and judge the working time of the prototype to charge it in time. The DC-DC module converts the voltage of the lithium battery (8.4 V) to 5 V and 3.3 V for peripheral devices and MCU. In addition, the DC-DC module isolates the interference of strong electrical signals on weak signals and realizes circuit voltage stabilization. The communication module is placed in the sealed cabin of the prototype, and a 433 Hz wireless module with high-speed data transmission is used to feed back the status information of the prototype in real-time. The wireless module uses Direct Sequence Spread Spectrum (DSSS) technology for communication, which has a strong anti-interference ability and can realize two-way transmission of communication data at 2 m underwater. The sensors are composed of IMU, depth sensor, and UWB location module. The IMU can obtain the attitude angle of the prototype, and communicate with the MCU by serial port. The depth sensor exchanges data with the MCU through the IIC protocols, and feeds back the depth information of the prototype, with an accuracy resolution of 2 mm. The UWB location module sends the position information of the robotic fish to the MCU by serial port, which can roughly estimate the position of the prototype, and the positioning error is 10 cm.

### 3.2. CPG Controller

After millions of years of evolution, biological fishes have fully adapted to the underwater environment with various characteristics. The interaction between the muscle-driven multifins achieves efficient, fast, and reliable swimming underwater. One of the design goals of the robotic fish is to mimic the swimming of a biological fish to achieve efficient locomotion similar to its bionic counterpart. By studying the principles of rhythmic movement in spinal cord animals, researchers found that CPG can generate rhythmic signals without the need for advanced nerve centers and feedback information [39]. As a rhythmic motion control method, CPG can generate stable rhythmic signals for controlling the movement of limbs and other related body parts. It has strong adaptability and robustness which is suitable for the motion control of robots [40,41]. With decades of development, artificial CPG controllers have been widely studied and applied to various biomimetic robots, such as four-legged robots [42,43], hexapod robots [44], amphibious robots [40,45], snake robots [46], and robotic fish [47,48]. Ichthyological studies have proven that the movements of the fins and bodies of fish are generated by the periodic activities of the central nervous system. Therefore, the researchers used various forms of CPG controllers to control the swimming gait of different swimming types of bionic robotic fish. Ichthyological studies have proven that the movements of the fins and bodies of fish are caused by the periodic activities of the central nervous system. Therefore, the researchers used various CPG controllers to control the swimming patterns of different types of bionic robotic fish [8]. There are three main CPG models applied to the bionic control of robotic fish: the neuron oscillator model [49], the recursive oscillator model [50], and the phase oscillator model [47]. The phase oscillator model has well-defined parameters such as frequency, amplitude, and phase lag, and enables asymmetric flapping of the pectoral and caudal fins. Therefore, we adopt the phase oscillator model for the biomimetic control of the prototype. The fins of the prototype are driven by six servomotors, corresponding to two pectoral fins and a caudal fin, respectively, and each servo motor is controlled by a phase oscillator unit. The six DOFs of the movement of the pectoral and caudal fins of the robotic fish correspond to six artificial CPG units, and the connection between the CPG units is realized by phase lags and coupling parameters. The artificial CPG topological network designed with 6 CPG units based on the phase oscillator model is shown in Figure 4b. The constructed phase oscillator model of the i-th CPG unit is as follows:

(2) $$\nu_{i}=\left[\frac{2\beta_{i}-1}{2\beta_{i}(1-\beta_{i})(1+e^{-k_{\nu i}{\dot{\theta }}_{i}})}+\frac{1}{2\beta_{i}}\right]\frac{1}{T_{i}}$$

(3) $${\ddot{r}}_{i}=a_{i}\left[\frac{a_{i}}{4}\left(R_{i}-r_{i}\right)-{\dot{r}}_{i}\right]$$

(4) $${\ddot{x}}_{i}=b_{i}\left[\frac{b_{i}}{4}\left(X_{i}-x_{i}\right)-{\dot{x}}_{i}\right]$$

(5) $$\Delta {\ddot{\varphi }}_{tij}=c_{ij}\left(\frac{c_{ij}}{4}\left(\Delta \varphi_{ij}-\Delta \varphi_{tij}\right)-\Delta {\dot{\varphi }}_{tij}\right)$$

(6) $${\dot{\phi }}_{i}=2\pi v_{i}+\sum_{i,j=1}^{6}\omega_{ij}\sin\left(\phi_{j}-\phi_{i}-\Delta \varphi_{tij}\right)$$

(7) $$\theta_{i}=x_{i}+r_{i}\sin\phi_{i}$$

The above model equations are the frequency continuity transition equation, amplitude equation, amplitude offset equation, desired phase lag continuity transition equation, phase equation, and output equation, and the corresponding parameters are shown in Table 2.

The driving servomotors of the pectoral and caudal fins have two directions of rotation, the counterclockwise direction is set as the up-beat of the fins, and the clockwise direction is set as the down-beat. Therefore, the designed frequency continuity transition equation makes the pectoral–caudal fins of the prototype have time-asymmetric flapping characteristics so that the up-beat and down-beat times of fins are not equal, that is, the up-beat frequency is different from the down-beat frequency. Judging whether the pectoral and caudal fins are in the upstroke or downstroke according to the positive and negative swing speeds of fins, the time asymmetric flapping of the pectoral and caudal fins is realized by setting different up-beat frequencies and down-beat frequencies, and the judgment method is formulated as:

(8) $$\{\begin{array}{ll} v_{i}=v_{i\_up}, & {\dot{\theta }}_{i}\geq 0\\ v_{i}=v_{i\_down}, & {\dot{\theta }}_{i}<0 \end{array}$$

where $v_{i\_up}$ is the up-beat frequency, $v_{i\_down}$ is the down-beat frequency of fins, and ${\dot{\theta }}_{i}$ is the angular velocity of the driving servomotors. ${\dot{\theta }}_{i}\geq 0$ represents the up-beat of fins, and ${\dot{\theta }}_{i}<0$ represents the down-beat of fins. In order to vividly describe the time asymmetry characteristics in the beating process of pectoral and caudal fins, the time asymmetry coefficient $\beta_{i}$ is introduced, which is defined as the ratio of fins’ up-beat time to the beating period. Therefore, the frequency can be expressed as:

(9) $$\{\begin{array}{l} v_{i}=v_{i\_up}=\frac{1}{2\beta_{i}T_{i}},\;\;\;{\dot{\theta }}_{i}\geq 0\\ v_{i}=v_{i\_down}=\frac{1}{2(1-\beta_{i})T_{i}},\;\;\;{\dot{\theta }}_{i}<0 \end{array}$$

where $\beta_{i}$ is the time asymmetric coefficient, $T_{i}$ is the swing period, $(1-\beta_{i})T_{i}$ is the up-beat time, and $\beta_{i}T_{i}$ is the down-beat time. However, due to the assignment of frequency segments, sudden changes in the frequency will cause sudden phase changes, which in turn will cause a jump in the output angle, resulting in an unsmooth swing of the pectoral and caudal fins. In order to avoid the influence of frequency mutation, the designed frequency continuity transition equation is shown in Equation (2).

In the CPG topology network based on the phase oscillator model, there is a synchronous steady-state vibration feature between any two oscillator units, and the phase lag between the steady-state vibration is determined by the desired phase lag. In the case of steady-state vibration between the two CPG units, the desired phase lag between two oscillator units can be expressed as:

(10) $$\Delta \varphi_{ij}=\phi_{j}-\phi_{i}$$

where $\phi_{i}$ and $\phi_{j}$ are the phases of the oscillator unit i and unit j, respectively. $\Delta \varphi_{ij}$ represents the coupled motion relationship between unit i and unit j. If $\Delta \varphi_{ij}>0$, then the oscillator unit j is in phase ahead of the oscillator unit i, and vice versa. Obviously, in the case of synchronized steady-state vibrations between units, $\Delta \varphi_{ij}=-\Delta \varphi_{ji}$ can be derived. For the CPG topology network in Figure 4b, its desired phase lag matrix Δφ is expressed as:

$$\Delta \varphi =\left[\begin{array}{cccccc} 0 & \Delta \varphi_{12} & 0 & \Delta \varphi_{14} & 0 & 0\\ \Delta \varphi_{21} & 0 & \Delta \varphi_{23} & 0 & 0 & 0\\ 0 & \Delta \varphi_{32} & 0 & 0 & \Delta \varphi_{35} & \Delta \varphi_{36}\\ \Delta \varphi_{41} & 0 & 0 & 0 & \Delta \varphi_{45} & 0\\ 0 & 0 & \Delta \varphi_{53} & \Delta \varphi_{54} & 0 & 0\\ 0 & 0 & \Delta \varphi_{63} & 0 & 0 & 0 \end{array}\right]$$

Since the synchronous steady-state vibration satisfies $\Delta \varphi_{ij}=-\Delta \varphi_{ji}$, there are only five independent variable parameters among the 12 desired phase lag parameters, and we select $\Delta \varphi_{12}$, $\Delta \varphi_{14}$, $\Delta \varphi_{45}$, $\Delta \varphi_{23}$ and $\Delta \varphi_{36}$ as the desired phase lag parameters control variables.

In order to determine the constant coefficient parameters in the CPG network, their values are changed and adjusted experimentally to obtain the appropriate values of the coupling coefficient between the oscillator units. Meanwhile, for the purpose of simplifying the control parameters, the coupling coefficient is set to two, the frequency transition coefficient is set to one, the amplitude and offset gain coefficients are set to 20, and the desired phase lag gain is set to 20. That is, $\omega_{ij}=2$ (CPG units topologically connected) or $\omega_{ij}=0$ (CPG units not topologically connected), $k_{vi}=1$, $a_{i}=b_{i}=20$ and $c_{ij}=20$. Due to the characteristics of the phase oscillator model, the oscillation frequency of the oscillation units with the topological connection relationship must be equal, so that the phase lag between the units is valid. If there is no coupling between the pectoral fins and the caudal fin, then the desired phase lags $\Delta \varphi_{23}$ and $\Delta \varphi_{35}$ are equal to 0. To decouple the topological relationship of the pectoral fin units and caudal fin unit, the coupling coefficients of the pectoral fin units and caudal fin unit need to be set to 0, that is, $\omega_{23}=\omega_{35}=0$. When only the phase relationship between pectoral fins is considered, the desired phase lag control variables are $\Delta \varphi_{12}$, $\Delta \varphi_{14}$ and $\Delta \varphi_{45}$. In this case, the diversification and flexibility of the swimming patterns of the prototype are realized by setting different oscillation frequencies for the pectoral and caudal fin units.

## 4. Hydrodynamic Experimental Results and Analysis

In the early work of our lab [28,51], we used the one/two-way FSI numerical analysis method to analyze the key parameters of the prototype. Based on the above simulation analysis, we built a hydrodynamic experiment environment for the robotic fish. The simulation results are verified by experiments, and the characteristic parameters of the pectoral and caudal fins of the prototype are analyzed. Based on the analysis of hydrodynamic experiments, the optimal parameters are selected to guide the design of the prototype, so as to improve the swimming performance of the prototype in the case of synergistic interaction between pectoral fins and the caudal fin.

### 4.1. Hydrodynamic Experimental Testing Environment

**Figure 5:**
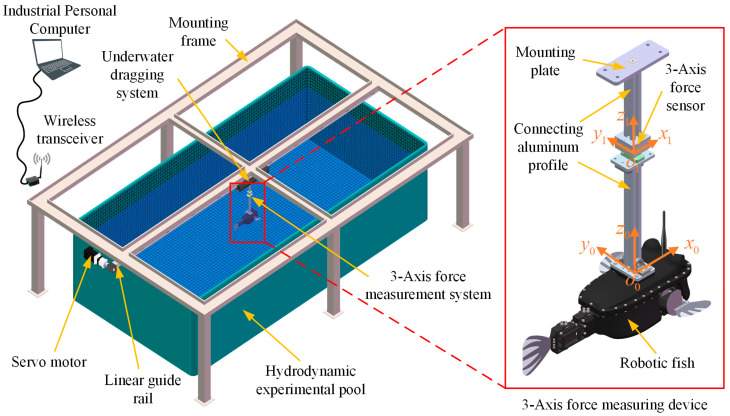
Schematic diagram of the hydrodynamic experimental platform.

In order to achieve the best state of swimming for the prototype, it is necessary to analyze the effects of parameters such as amplitude, frequency, offset angle, desired phase lag, and time asymmetric coefficient in the CPG controller based on the phase oscillator model on the swimming performance of the robotic fish. Based on the numerical simulation analysis of our work [28,51], we designed and built the swimming performance testing platform of the robotic fish, and further explored the inner relationship between these parameters by experimental device method, so as to select the best parameters to guide the movement of the robotic fish. Obviously, thrust and lift are the main factors affecting the propulsion capability of the robotic fish prototype, while the turning of the robotic fish is mainly realized by the lateral force generated by the coordinated swing of the pectoral fins and caudal fin. Therefore, the effects of these parameters on the thrust, lift, and lateral force during the swimming of the robotic fish were determined experimentally. The effects of different CPG parameters on the swimming performance of the prototype were analyzed by the single variable method. That is, select typical parameters, change a single variable parameter, and measure the thrust and lift force of the robotic fish with different parameters on the experimental platform. The testing environment of the hydrodynamic experiments is shown in Figure 5, which is mainly composed of four parts: the experimental pool, the towing guide rail, the force-measuring system, and the robotic fish.

The force-measuring system is connected to the drag rail and can move along the rail, and the speed and position can be adjusted by the AC servomotor. To reduce the interference of the water flow fluctuation on the pool wall to the robotic fish, the drag guide rail is installed in the center of the pool, 1400 mm away from the pool walls on both sides. Also, for the sake of reducing the impact of the pool bottom and the water surface, the center of gravity of the robot fish is located at the center of the pool, with a depth of about 500 mm. The force-measuring system utilizes a three-axis force sensor to measure the thrust, lateral force, and lift force on the prototype. The robot fish is connected to the sensor through the mounting base and hangs in the pool. The force was measured by using a three-axis force sensor (50 N, FC3D60, Shanghai Forcechina Measurement Technology Co., Ltd. Shanghai, China), and the measuring data were recorded at a frequency of 125 Hz using a measuring amplifier (GSV-4USBSubD37, ME-Meßsysteme GmbH, Hennigsdorf, Germany). The coordinate axis of the three-axis force sensor is in the same direction as the coordinate axis of the prototype, and its *z*-axis coincides with the *z*-axis direction of the robot fish. The origin of the coordinate system of the robotic fish is located at the center of mass. The three-axis force-measuring system is shown in Figure 5, which can measure the force in the forward direction (*x*-axis, thrust force), gravity direction (*z*-axis, lift force), and lateral direction (*y*-axis, lateral force) of the prototype.

### 4.2. Hydrodynamic Measuring Method

**Figure 6:**
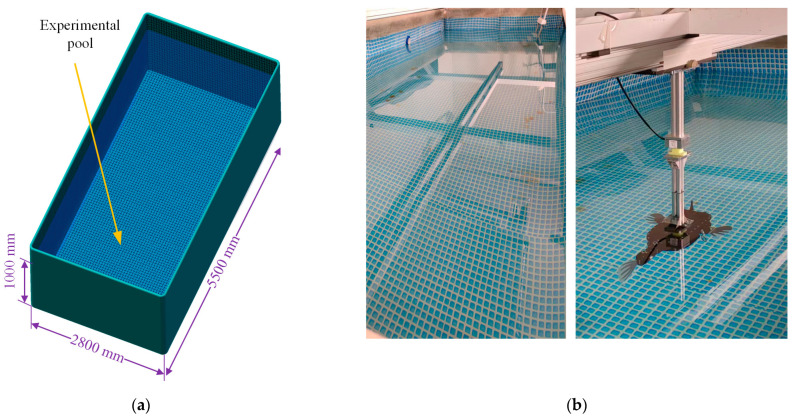
Experimental pool and 3-axis Force Measurement System. (**a**) Schematic diagram of the swimming pool; (**b**) Actual swimming pool and 3-axis force measuring device.

To simulate the hydrodynamic swimming performance of the prototype in the actual underwater environment, the three-axis force-measuring device composed of the sensor and the prototype is fixed on the slider of the towing system. Meanwhile, the servomotor of the dragging system is controlled to move the prototype to the center of the pool, so as to realize the measurement and analysis of the force generated by the synergistic interaction of the pectoral fins and caudal fin in the still underwater environment. The swimming performance of the prototype is mainly affected by the thrust and lift force generated by the synergistic interaction between the pectoral and caudal fins, and the lateral force mainly affects the turning performance of the prototype. According to the previous work [28], the thickness of the carbon fiber skeleton of the pectoral fins and caudal fin of the prototype is 0.2 mm and 0.3 mm, respectively. The hydrodynamic performance of the prototype with different CPG parameters was tested by using the built hydrodynamic testing platform, that is, the three-axis forces generated by the swimming of the prototype in the still underwater environment are measured and analyzed, and the CPG parameters with better hydrodynamic characteristics are determined. Figure 6 shows the experimental environment for testing the hydrodynamic performance of the prototype. Based on the testing system, the t3-axis forces of the prototype under the independent swing of the caudal fin, the independent swing of the pectoral fins, and the synergistic interaction of the pectoral and caudal fins are measured, respectively. Furthermore, the effects of the CPG parameters of the caudal fin, the parameters of the pectoral fins, and the synergistic interaction parameters of the pectoral fins and caudal fin on the hydrodynamic performance of the prototype are analyzed by means of the measured three-axis force data, so as to select better CPG control parameters.

### 4.3. Effects of the Caudal Fin Parameters on Propulsion Performance

In general, fish in the BCF mode relies on the swing of the caudal fin to form a backward propulsion wave to achieve efficient and fast movement, and their dorsal, anal, pectoral, and pelvic fins are utilized to assist propulsion and adjust posture. That is, the rapidity and maneuverability of the prototype in different swimming modes can be adjusted through the synergistic interaction of the caudal fin and the pectoral fins. The tail of the prototype accounts for a large proportion of the overall length, and the swing of the caudal fin plays a major role in the propelling process of the prototype, while the pectoral fins mainly affect the maneuverability of the prototype. Therefore, in this section, the effects of the pectoral fins and caudal fin are decoupled first, and the effects of the CPG parameters of the two-DOF tail joints under the action of the caudal fin on the propulsion performance of the prototype are considered. The center of mass of the two tail joints of the prototype is located on the same horizontal plane as that of the body, and the rotation axes of the two tail joints are both perpendicular to the horizontal plane, so the swing of the caudal fin can only provide force in the horizontal direction. Obviously, the caudal fin of the prototype swings symmetrically during straight-line swimming, so that the lateral force generated during the swing of the tail joint is balanced. Therefore, the amplitude offset angle of the two tail joints is set to 0, i.e., $X_{3}=X_{6}=0$. At the same time, the up-beat and down-beat frequencies of the caudal fin are equal, in order to avoid the difference in lateral force on both sides of the body caused by time asymmetrical flapping, that is, $v_{3,6\_up}=v_{3,6\_down}$. We use the hydrodynamic testing method in the still underwater environment to analyze the effects of the amplitude, phase lag, and frequency of the two-DOF tail joints on the propulsion performance, so as to select the CPG parameters with better propulsion performance. For the sake of avoiding interference with the pectoral fins, the amplitude and amplitude offset angle of the pectoral fins are set to 0, that is, $R_{1}$, $R_{2}$, $R_{4}$, $R_{5}$, $X_{1}$, $X_{2}$, $X_{4}$ and $X_{5}$ are all 0°.

#### 4.3.1. Effects of the Amplitude Parameter of the Caudal Fin on Propulsion Performance

**Figure 7:**
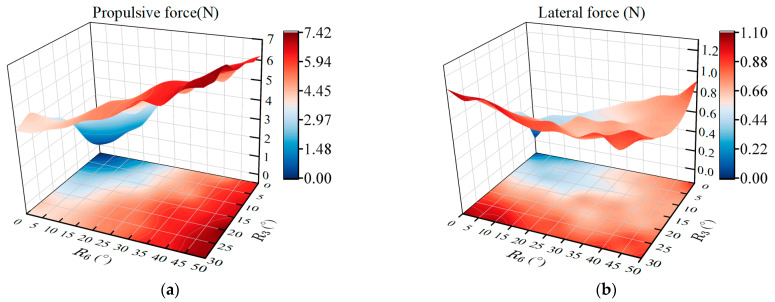
Effects of the amplitudes between the two caudal joints on propulsive performance. (**a**) Propulsive force; (**b**) Lateral force.

To analyze the effect of the amplitude of the tail joint three and joint six on the propulsion performance, the pectoral fin is set to stationary to avoid interference with the pectoral oscillation. Meanwhile, based on the data obtained from the FSI simulation analysis method, the better parameters in the simulation results are selected to set the frequency and phase lag of the two-DOF tail joints. The frequencies of the tail joints are set to 1 Hz and the phase lag is set to $-90^{{}^{\circ}}$, i.e., $v_{3}=v_{6}=1$ and $\Delta \varphi_{36}=-90^{{}^{\circ}}$. The amplitudes $R_{3}$ and $R_{6}$ of the joint three and joint six are set as variable parameters, where $R_{3}$ is taken from $0^{{}^{\circ}}$ to $30^{{}^{\circ}}$ and $R_{6}$ is taken from $0^{{}^{\circ}}$ to $50^{{}^{\circ}}$ with an interval of $5^{{}^{\circ}}$. After the caudal fin oscillation period was stabilized in each experiment, the sensor data were read, and the absolute values of the thrust ($F_{x}$) and lateral force ($F_{y}$) amplitudes are taken. The changes in thrust and lateral force for different combinations of two-tail joints are shown in Figure 7.

When the amplitude of the tail joints of the robotic fish increases, the swing amplitude of its caudal fin increases, and the thrust and lateral force generated by the prototype increase significantly. From the overall change trend, when the amplitude of joint three is fixed, the thrust and lateral force generated by the prototype increase with the increase of the amplitude of joint six. When the amplitude of joint six is fixed, the thrust and lateral force produced by the robotic fish are positively correlated with the amplitude of joint three. However, when the amplitude of joint three is $R_{3}=15^{{}^{\circ}}$, the lateral force generated by the prototype tends to decrease within the range of the amplitude of joint six as $15^{{}^{\circ}}∼20^{{}^{\circ}}$. The increase in thrust can increase the speed of the robotic fish, which is beneficial to realize the rapid movement of the prototype. The reduction of the lateral force can reduce the reciprocating swing in the heading direction, which is beneficial to the stability of the heading. Therefore, the selection of the amplitude should comprehensively consider the effect of thrust and lateral force. It is worth mentioning that, by observing the swimming of biological boxfish, we found that the maximum swing range of the caudal fin relative to the caudal peduncle is $30^{{}^{\circ}}∼50^{{}^{\circ}}$. At the same time, according to the thrust and lateral force measurement results in Figure 7, the amplitude of joint three is set as $15^{{}^{\circ}}$, and the amplitude of joint six has good propulsion performance under the conditions of $15^{{}^{\circ}}$ and $20^{{}^{\circ}}$, that is, $R_{3}=15^{{}^{\circ}}$, $R_{6}=15^{{}^{\circ}}$ or $R_{6}=20^{{}^{\circ}}$. There are two options for the amplitude of joint six. We will comprehensively analyze them in the subsequent phase lag and frequency hydrodynamic experiments to select better parameters.

#### 4.3.2. Effects of the Phase Lags of Two Caudal Fins on Propulsion Performance

**Figure 8:**
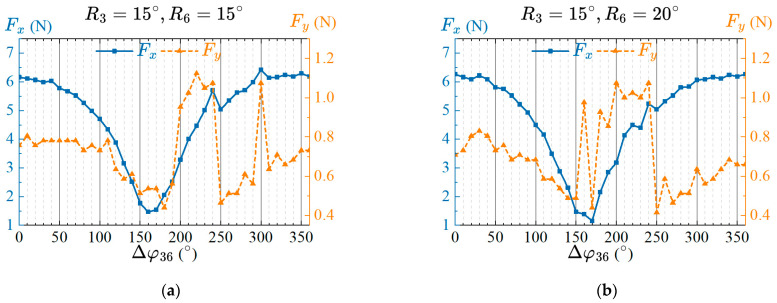
T Effects of the phase lags between the two caudal joints on propulsive performance for two amplitudes. (**a**) Amplitudes of the two caudal joints are $R_{3}=15^{{}^{\circ}}$, $R_{6}=15^{{}^{\circ}}$; (**b**) Amplitudes of the two caudal joints are $R_{3}=15^{{}^{\circ}}$, $R_{6}=20^{{}^{\circ}}$.

The amplitude of joint three is set to $15^{{}^{\circ}}$, and the effect of phase lag on propulsion performance is analyzed under the condition that the amplitude of joint six is set to $15^{{}^{\circ}}$ or $20^{{}^{\circ}}$. Set the phase lag $\Delta \varphi_{36}$ between the two tail joints as a variable parameter ranging from $0^{{}^{\circ}}$ to $360^{{}^{\circ}}$ with an interval $10^{{}^{\circ}}$. After swinging is stable, the thrust and lateral force of the prototype are measured, and the results are shown in Figure 8.

When the amplitude of joint six is $15^{{}^{\circ}}$ or $20^{{}^{\circ}}$, the thrust generated by the caudal fin to the prototype decreases with the increase of the phase lag $\Delta \varphi_{36}$, and when $\Delta \varphi_{36}$ is $170^{{}^{\circ}}$, the thrust is minimized. Subsequently, the thrust further increases with the increase of the phase lag, is maximized when $\Delta \varphi_{36}$ is $300^{{}^{\circ}}$, and gradually stabilizes. The lateral force first decreases slowly with the increase of the phase lag, then increases rapidly, and finally transitions from a rapid decrease to a slow increase. Considering that the thrust directly affects the swimming speed of the robotic fish, and the lateral force will cause the swing of the prototype’s heading direction, the selection of phase lag should make larger thrust and smaller lateral force. Therefore, when the phase lag $\Delta \varphi_{36}$ is $270^{{}^{\circ}}$ or $290^{{}^{\circ}}$, the propulsion performance of the robotic fish is ideal. In order to determine the better CPG parameters of the phase lags, we will analyze the propulsion performance of the prototype with different frequency parameters based on four parameter combinations of two amplitudes and phase lags.

#### 4.3.3. Effects of the Frequency Parameter of the Caudal Fin on Propulsion Performance

**Figure 9:**
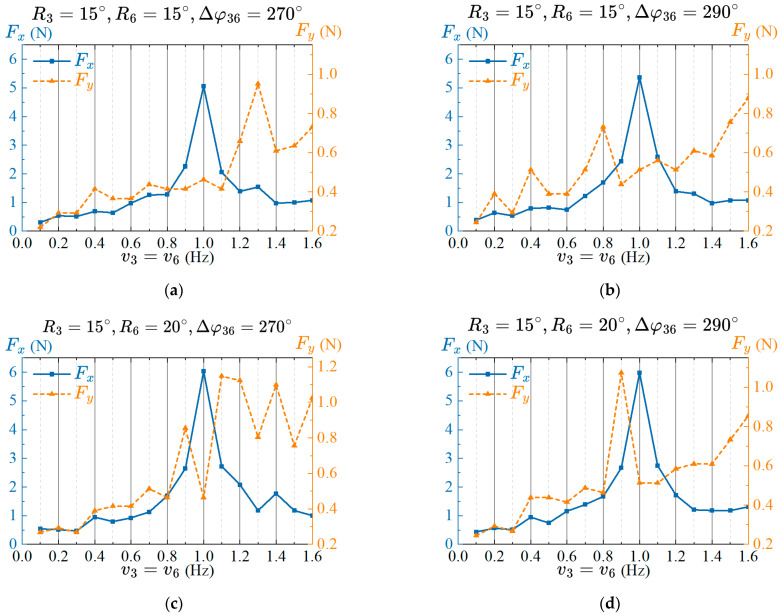
Effects of the frequency on propulsive performance for two amplitudes and phase lags. (**a**) Amplitude and phase lags are $R_{3}=15^{{}^{\circ}}$, $R_{6}=15^{{}^{\circ}}$, $\Delta \varphi_{36}=270^{{}^{\circ}}$; (**b**) Amplitude and phase lags are $R_{3}=15^{{}^{\circ}}$, $R_{6}=15^{{}^{\circ}}$, $\Delta \varphi_{36}=290^{{}^{\circ}}$; (**c**) Amplitude and phase lags are $R_{3}=15^{{}^{\circ}}$, $R_{6}=20^{{}^{\circ}}$, $\Delta \varphi_{36}=270^{{}^{\circ}}$; (**d**) Amplitude and phase lags are $R_{3}=15^{{}^{\circ}}$, $R_{6}=20^{{}^{\circ}}$,$\Delta \varphi_{36}=290^{{}^{\circ}}$.

Based on the analysis of the optimal CPG parameters of amplitude and phase lag in the previous two sections, this section explores the effect of the frequency of the caudal fin on the propulsion performance and selects the best combination parameters. Limited by the drive capability of the servomotor and the structural strength of the mechanism, the excessive frequency will cause vibrations in the prototype, so the upper limit of the frequency is set to 1.6 Hz. Set the frequency of the caudal fin as a variable parameter, with a frequency range of 0.1 Hz to 1.6 Hz with an interval of 0.1 Hz. The propulsion performance testing method is the same as in the previous sections. The data of thrust and lateral force caused by frequency changes under the four combinations of amplitudes and phase lags are measured. The results are shown in Figure 9.

The variation trend of the thrust with the frequency under the four different combinations is roughly the same, and the thrust increases first with the increase of the frequency, reaches the maximum value at 1 Hz, and then decreases. The overall variation trend of lateral force increases with frequency, but has a smaller value at 1 Hz. Therefore, when the frequency of the caudal fin is 1 Hz, the propulsion performance of the robotic fish is ideal, with greater propulsion force and smaller lateral force. Combining the thrust and lateral force generated by the previous amplitude and phase lag, and simultaneously analyzing the thrust and lateral force of the prototype under the condition that the frequency is 1 Hz, the amplitude, phase lag, and frequency of the caudal fin with better propulsion performance are selected. The CPG parameters are $R_{3}=15^{{}^{\circ}}$, $R_{6}=20^{{}^{\circ}}$, $\Delta \varphi_{36}=270^{{}^{\circ}}$, $v_{3}=1\;\mathrm{Hz}\;$and $v_{6}=1\;\mathrm{Hz}$, respectively.

### 4.4. Effects of the Pectoral Fin Parameters on Propulsion Performance

The joint one and joint four of pectoral fins are defined as the twisting of the fin base, and joint two and joint five are defined as the flapping of the fin surface. To explore the effects of the CPG parameters of the pectoral fins on the propulsion performance of the prototype, we adopt the control variable method to measure the thrust and lift force generated by different CPG parameters. The two-DOF tail joints rest in a central position and remain fixed to avoid the influence of the caudal fin on the pectoral fins. Since the pectoral joints on both sides of the prototype are symmetrical, we set $R_{1}=R_{4}$, $R_{2}=R_{5}$. Meanwhile, in order to eliminate the lateral force generated by the pectoral fins on both sides, joint one and joint four need to move in the same direction or the opposite direction when the prototype is propelled forward, that is, $\Delta \varphi_{14}=0^{{}^{\circ}}$ or $\Delta \varphi_{14}=180^{{}^{\circ}}$. In addition, due to the symmetrical arrangement of the pectoral fins on both sides, setting them to swing symmetrically can eliminate the lateral force. Therefore, we can choose to move one side of the pectoral fin and keep the other side still and only measure the thrust and lift force generated by the movement of one side of the pectoral fin. Next, we will take the right pectoral joint four and joint five fins as an example to measure and analyze the effect of different pectoral CPG parameters (amplitude, bias, phase lag, frequency, and time asymmetric coefficient) on the propulsion performance of the prototype.

#### 4.4.1. Effects of the Amplitudes of Pectoral Fin Twisting and Flapping on Propulsion Performance

**Figure 10:**
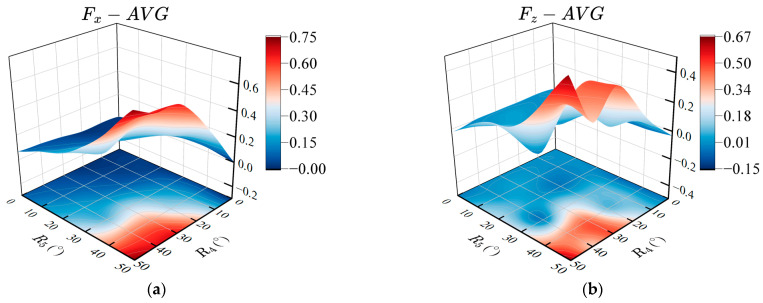
Effects of the amplitudes of twisting and flapping on propulsion performance. (**a**) Average propulsive force; (**b**) Average lift force.

In order to explore the effect of the amplitude of pectoral twisting and flapping on the propulsion performance of the robotic fish, it is necessary to utilize the amplitude as a variable parameter, that is, to measure the thrust and lift force of the prototype with different values of $R_{1}$ and $R_{2}$. We set the amplitude offset angle to $0^{{}^{\circ}}$, and the phase lag between pectoral joint four and joint five to be $90^{{}^{\circ}}$, namely, $X_{4}=0^{{}^{\circ}}\;$, $X_{5}=0^{{}^{\circ}}\;$, and $\Delta \varphi_{45}=90^{{}^{\circ}}$. Based on the observation of the pectoral swinging process of the biological boxfish, the amplitudes of the twisting and flapping range from $0^{{}^{\circ}}$ to $50^{{}^{\circ}}$, and the interval is $10^{{}^{\circ}}$. The average thrust and lift forces after the stable swinging are measured and the results are shown in Figure 10.

Figure 10a shows the relationship between the amplitude of twisting and flapping of pectoral fins and the average thrust. Increasing the amplitude of twisting and flapping can increase the average thrust of the prototype. In the case of the same twisting amplitude, the greater the flapping amplitude, the greater the average thrust. Meanwhile, the average thrust shows a positive correlation with the twisting amplitude for the same flapping amplitude. Figure 10b shows the relationship between the twisting and flapping amplitudes and the average lift force. Different values of amplitude will subject the prototype to positive or negative lift force. Overall, there is a positive correlation between the twisting and flapping amplitudes and the average lift force. Considering the average thrust and lift force generated by different twisting and flapping amplitudes comprehensively, select the combination of amplitudes whose average lift is approximately zero and the average thrust is relatively large, that is, the optimal amplitude parameters are $R_{1}=40^{{}^{\circ}}$ and $R_{2}=30^{{}^{\circ}}$.

#### 4.4.2. Effects of the Offset Angles between Twisting and Flapping of the Pectoral Fins on Propulsion Performance

**Figure 11:**
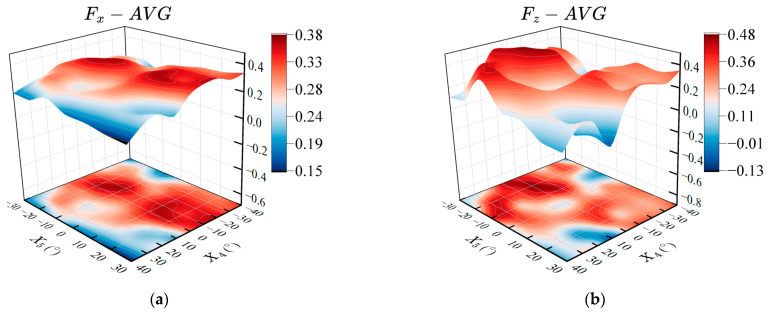
Effects of the offset angles between pectoral fin twisting and flapping on propulsion performance. (**a**) Average propulsive force; (**b**) Average lift force.

The amplitude offset angle changes the angle of attack of the fin surface, which mainly affects the upward and downward movements of the prototype. In order to explore the effect of the amplitude offset angle of twisting and flapping on the propulsion performance of the prototype, the amplitude offset angles of the fin base and fin surface are used as the variable parameters. Based on the amplitude parameters in the previous section, we set the other parameters as: $R_{4}=40^{{}^{\circ}}$, $R_{5}=30^{{}^{\circ}}$, and $\Delta \varphi_{45}=90^{{}^{\circ}}$. The twisting amplitude offset angle of the fin base ranges from $-40^{{}^{\circ}}$ to $40^{{}^{\circ}}$, and the flapping offset angle of the fin surface ranges from $-30^{{}^{\circ}}$ to $30^{{}^{\circ}}$, with an interval of $10^{{}^{\circ}}$. The average thrust within three periods after the stable swinging of the pectoral fin is measured, and the results obtained together with the amplitude offset angle are shown in Figure 11.

As shown in Figure 11a, compared with the amplitude offset angle of flapping, the twisting offset angle of the pectoral fin has a greater impact on the average thrust of the robotic fish, and a larger average thrust can be generated within the range of the twisting offset angle from $-40^{{}^{\circ}}$ to $0^{{}^{\circ}}$. Figure 11b shows the effect of different twisting and flapping amplitude offset angles on the average lift force. Changing the amplitude offset angle of flapping can significantly affect the average lift force. When the offset angles of twisting and flapping are equal, the pectoral fins of the robot fish exhibit the movement characteristics of lift-based mode and can maintain a large propulsion force in a still underwater environment, and the average lift force is close to zero. That is, the lift forces generated by the swing of the pectoral fins are counteracted by each other during one period. When the offset angles of twisting and flapping are $X_{4}=40^{{}^{\circ}}$, $X_{5}=30^{{}^{\circ}}$ or $X_{4}=-40^{{}^{\circ}}$, $X_{5}=-30^{{}^{\circ}}$, the average lift force is approximately zero, and it can also generate a certain propulsion force, which is in line with the movement characteristics of the drag-based mode [52].

#### 4.4.3. Effects of the Phase Lag between Pectoral Fin Twisting and Flapping on Propulsion Performance

**Figure 12:**
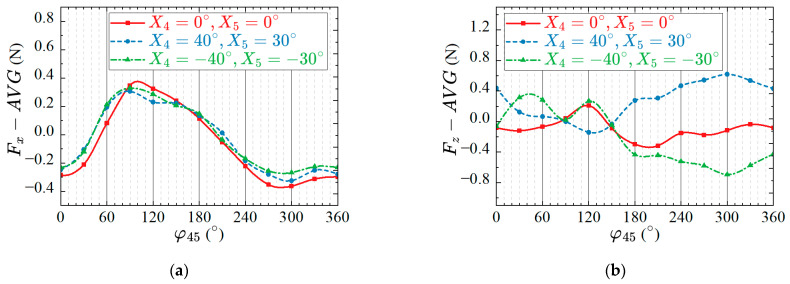
Effects of the phase lag between twisting and flapping on propulsion performance. (**a**) Average propulsive force; (**b**) Average lift force.

In order to quantitatively analyze the effects of the phase lag between twisting and flapping on the propulsion performance of robotic fish, we set other parameters as $R_{4}=40^{{}^{\circ}}$, $R_{5}=30^{{}^{\circ}}$, $v_{4}=v_{5}=1\mathrm{Hz}$. The phase lag $\Delta \varphi_{45}$ between twisting and flapping is taken as the control variable, the value ranges from $0^{{}^{\circ}}$ to $360^{{}^{\circ}}$, with an interval of $30^{{}^{\circ}}$, and the average thrust and lift force of the prototype in lift-based mode and drag-based mode are measured, respectively. After the value of the phase lag is changed each time, the average thrust and lift forces are measured after swinging stably. The relationship between the forces and the phase lag is shown in Figure 12.

It can be seen from Figure 12a that in both the lift-based mode or the drag-based mode, the average propulsion value of the robotic fish shows positive and negative changes, indicating that different phase lags can change the propulsion direction of the prototype. Meanwhile, the two modes can generate greater propulsion when the phase lag is near $90^{{}^{\circ}}$. Figure 12b shows the variation of the average lift force with the phase lag in the lift-based mode and drag-based mode of the prototype. Although the average lift curve of the prototype in the lift-based mode has some fluctuations with the change of the phase lag, the overall effect is not significant. It shows that the robotic fish can swim forward and backward by changing the phase lag between the twisting and flapping of the pectoral fin in the lift-based mode, without having a large impact on the pitch. In the drag-based mode, the average lift force of the prototype in still underwater is greatly affected by the change of the phase lag, and a large average lift force can be generated within a specific range, and the average lift force shows a certain positive and negative difference. Therefore, the pitching of the prototype can be realized by changing the phase lag $\Delta \varphi_{45}$ in the drag-based mode. In other words, by choosing the lift-based mode or the drag-based mode, changing the phase lag $\Delta \varphi_{45}$ can realize the forward, backward and pitching swimming of the robotic fish.

#### 4.4.4. Effects of the Frequency of Pectoral Fin on Propulsion Performance

**Figure 13:**
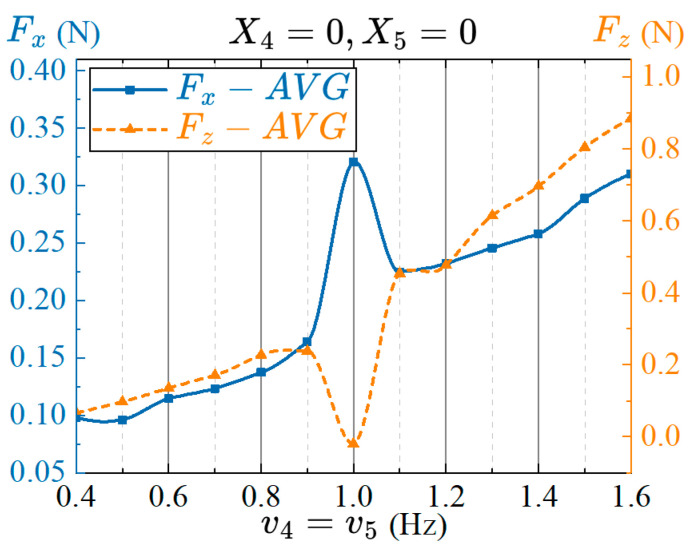
Effect of the frequency on propulsion performance in lift-based mode.

**Figure 14:**
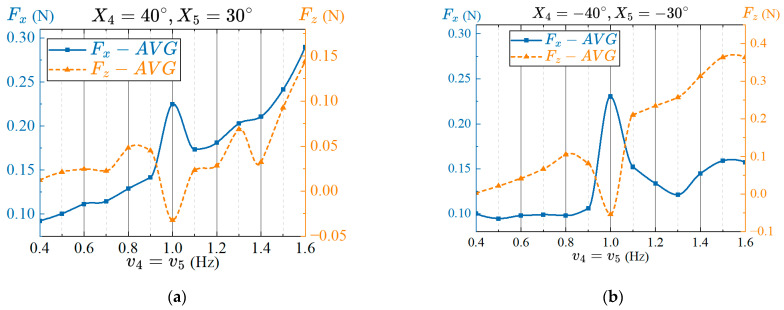
Effects of the frequency on propulsion performance in drag-based mode. (**a**) $X_{4}=40^{{}^{\circ}}$, $X_{5}=30^{{}^{\circ}}$; (**b**) $X_{4}=-40^{{}^{\circ}}$, $X_{5}=-30^{{}^{\circ}}$.

The frequency determines how fast the pectoral fins swing, which in turn affects the performance of the prototype. If the swing frequency of the pectoral fins is too slow, the force exerted by the fins on the water flow will be too small, which will affect the transmission of propulsion waves and cannot form sufficient propulsion. Swinging too fast will cause excessive water flow disturbance, resulting in viscous resistance and affecting propulsion efficiency. Furthermore, high-frequency swing of pectoral fins will also affect the stability of the robotic fish during swimming. Therefore, exploring the effects of the frequency of swing on the propulsion performance of the prototype, and choosing an appropriate frequency is conducive to improving the propulsion performance and stability of the robotic fish. Set the frequency parameter as the control variable, set the twisting and flapping amplitudes as $R_{4}=40^{{}^{\circ}}$ and $R_{5}=30^{{}^{\circ}}$, respectively. Set the phase lag between twisting and flapping as $\Delta \varphi_{45}=90^{{}^{\circ}}$. If the frequency is too fast, the servomotor will vibrate, so the frequency ranges from 0.5 Hz to 1.6 Hz, and the interval is 0.1 Hz. The average thrust in several cycles after the stable swing of the pectoral fin are measured, and the results together with the frequency changes are shown in Figure 13 and Figure 14.

The effects of swing frequency on the average thrust and lift force in lift-based mode is shown in Figure 13. It can be seen from the figure that the average thrust of the prototype is the largest at 1 Hz, and the average lift force is zero, which has the best propulsion performance. It is worth noting that, except for the data at 1 Hz, the average thrust and lift force generated by pectoral fin increase with the increase of frequency, and present a certain approximate linear relationship. That is, the swimming speed of the prototype can be adjusted by appropriately changing the frequency of the pectoral fin.

In drag-based mode, the relationship between the frequency of the pectoral fins and the average forces is shown in Figure 14. It can be seen that the propulsion performance at 1 Hz is optimal regardless of the amplitude offset angle is $X_{4}=40^{{}^{\circ}}$, $X_{5}=30^{{}^{\circ}}$ or $X_{4}=-40^{{}^{\circ}}$, $X_{5}=-30^{{}^{\circ}}$, with a larger average thrust and an average lift force that is approximately zero. The difference is that, under the condition of the amplitude offset angle $X_{4}=40^{{}^{\circ}}$, $X_{5}=30^{{}^{\circ}}$, the average thrust data at 1 Hz are ignored, and the average thrust increases approximately linearly with frequency. In the case of the amplitude offset angle $X_{4}=-40^{{}^{\circ}}$, $X_{5}=-30^{{}^{\circ}}$, the average lift force increases approximately linearly with the frequency after removing the average lift force data at 1 Hz. Therefore, when the robotic fish swims in the drag-based mode, the thrust or lift force can be changed by selecting different amplitude offset angles and adjusting the frequency of pectoral twisting and flapping, so as to realize the adjustment of the propulsion speed and upward movement of the robotic fish.

#### 4.4.5. Effect of the Time Asymmetric Flapping of Pectoral on Propulsion Performance

**Figure 15:**
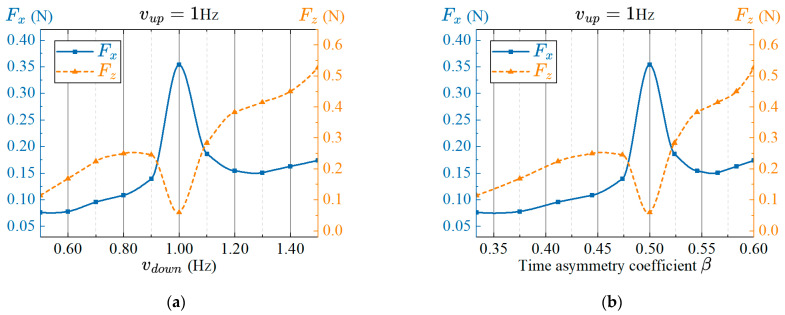
Effects of the time asymmetric flapping on propulsion performance. (**a**) Frequency; (**b**) Time asymmetric coefficient.

Through the observation of the biological boxfish, we found that the times of the power stroke and recovery stroke of the pectoral fins are not exactly the same when the boxfish adjusts its posture, which is called time asymmetric flapping characteristics. In order to study the influence of the time asymmetric flapping characteristics of pectoral fins on propulsion performance, we divide the swing stroke into power stroke and recovery stroke and change the time asymmetry coefficient to change the frequency of different strokes. The frequency of the power stroke is expressed as $v_{up}$, and the frequency of the recovery stroke is $v_{down}$. The amplitudes of twisting and flapping are $R_{1}=40^{{}^{\circ}}$ and $R_{2}=30^{{}^{\circ}}$, respectively. The phase lag between twisting and flapping is $\Delta \varphi_{12}=90^{{}^{\circ}}$. From the results in the previous section, it can be seen that the best frequency of pectoral fins is 1 Hz. Therefore, set the frequency of the power stroke to 1 Hz, and change the frequency of the recovery stroke to realize the change of the time asymmetry coefficient. The frequency of the recovery stroke ranges from 0.5 Hz to 1.5 Hz with an interval of 0.1 Hz. The average thrust in several cycles after the stable swing of the pectoral fin are measured. The results are shown in Figure 15. The time asymmetry coefficient is formulated as:

(11) $$\beta =\frac{v_{down}}{v_{up}+v_{down}}$$

The time asymmetry coefficient increases with the frequency of the recovery stroke $v_{down}$. The experimental testing results show that the average thrust firstly increases and then decreases with the increase of the time asymmetric coefficient over time, and reaches the maximum value at β=0.5, and the thrust is more significant. The average lift force has a minimum value at β=0.5, which is close to zero. Except for the minimum value, the overall change trend of the average lift force increases with the increase of the time asymmetry coefficient. Overall, the time asymmetry coefficient has a certain influence on the average thrust and lift force. When β=0.5, that is, the time-symmetric flapping of the pectoral fins, the average propulsion is the largest and the lift force is small. Moreover, the average lift force increases significantly at β>0.5, indicating that the robotic fish can obtain positive lift force through the rapid recovery stroke of the pectoral fins. That is to say, the robot fish can realize the attitude control of floating by adjusting the time asymmetry coefficient β.

### 4.5. Effects of Synergistical Interaction between the Pectoral Fins and the Caudal Fin

In the previous sections, the average thrust and lift force of the prototype with different control parameters were measured under the independent action of the caudal fin and pectoral fins, and the effects of different control parameters on the average thrust and lift force of the robotic fish was analyzed. Different thrusts will not only change the swimming attitude of the prototype, such as forward, backward, and pitching swimming, but also affect the stability and maneuverability of the prototype during swimming. Hove et al. [53] observed the swimming process of the biological boxfish, and their results showed that the boxfish can stabilize itself and achieve flexible attitude changing by means of the synergistic interaction of multiple fins. The synergistic interaction between the pectoral fins and caudal fin will affect the stability of the prototype during swimming, and the control parameters of the interaction are the phase lag between the fin bases of the pectoral fins on both sides and the phase lag between the pectoral and caudal fins. that is, the effects of different values of $\Delta \varphi_{14}$ and $\Delta \varphi_{23}$ on the thrust performance of the prototype are analyzed. In addition, in the designed artificial CPG topological network, the pectoral and caudal fins have the same frequency. Therefore, we also measured and analyzed the effects of different frequencies on the propulsion performance in the case of synergistic interaction of pectoral fins and the caudal fin.

#### 4.5.1. Effects of the Phase Lags $\Delta \varphi_{14}$ and $\Delta \varphi_{23}$ on Propulsion Performance

**Figure 16:**
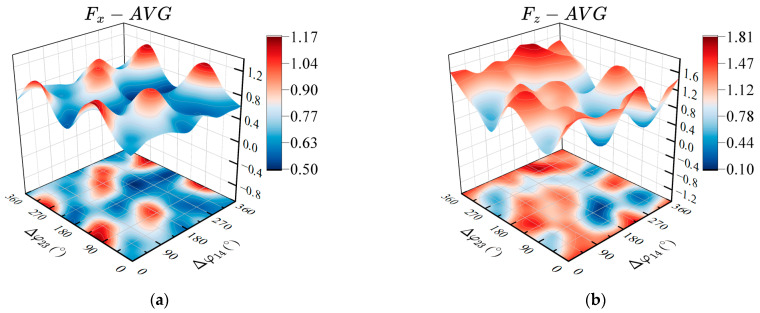
Effects of the phase lags on propulsion performance under the synergistical interaction between pectoral fins and caudal fin. (**a**) Average propulsive force; (**b**) Average lift force.

By measuring the average thrust and lift force generated by different phase relationships in the case of the synergistic interaction between pectoral fins and the caudal fin, the effects of the coupled phase relationship between the pectoral and caudal fins on the propulsion performance of the robotic fish were analyzed. We select the optimal control parameters measured by the previous experiments as: $R_{1}=R_{4}=40^{{}^{\circ}}$, $R_{2}=R_{5}=30^{{}^{\circ}}$, $R_{3}=15^{{}^{\circ}}$, $R_{6}=20^{{}^{\circ}}$, $\Delta \varphi_{12}=\Delta \varphi_{45}=90^{{}^{\circ}}$, $\Delta \varphi_{36}=270^{{}^{\circ}}$, $X_{i}=0^{{}^{\circ}}(i=1,\cdots ,6)$, $v_{i}=1\;\mathrm{Hz}(i=1,\cdots ,6)$. The twisting phase lag $\Delta \varphi_{14}$ between the left and right sides of the pectoral fins and the phase lag $\Delta \varphi_{23}$ between the flapping of the pectoral fins and the joint of the caudal fin are used as the control variables. The phase lags $\Delta \varphi_{14}$ and $\Delta \varphi_{23}$ range from $0^{{}^{\circ}}$ to $360^{{}^{\circ}}$, with an interval of $45^{{}^{\circ}}$. Then, we measure the average thrust and average lift force of the robotic fish during the three motion periods after the stable swing. The variation of the average thrust and lift force with the phase lags $\Delta \varphi_{14}$ and $\Delta \varphi_{23}$ is shown in Figure 16.

As shown in Figure 16a, the average thrust of the robotic fish is not greatly affected under the condition of different combinations of phase lags $\Delta \varphi_{14}$ and $\Delta \varphi_{23}$, and only a few specific combination values of phase lags can generate greater average thrust. From the aforementioned testing analysis propelled by dependent pectoral fins or dependent fin, it can be seen that the control parameters which have a greater impact on the thrust of the prototype are mainly amplitude and frequency. When the value of $\Delta \varphi_{14}$ is $0^{{}^{\circ}}$ or $180^{{}^{\circ}}$, that is, twisting joints of pectoral fins on both sides owing in-phase ($\Delta \varphi_{14}=0^{{}^{\circ}}$) or out of phase ($\Delta \varphi_{14}=180^{{}^{\circ}}$), the movement gait corresponding to the phase lag $\Delta \varphi_{23}$ between the pectoral fins and the caudal fin is $90^{{}^{\circ}}$ or $-90^{{}^{\circ}}$ can generate greater propulsion. Figure 16b shows the average lift force of the prototype with different combinations of phase lags. It can be seen from the figure that the average lift force is significantly affected by the combination of phase lags. When the phase lag $\Delta \varphi_{23}$ between pectoral fins and the caudal fin ranges from $90^{{}^{\circ}}$ to $180^{{}^{\circ}}$, the average lift force is generally larger. Moreover, the average lift force under the condition that the left and right pectoral fins are in-phase is greater than the lift force of the reversed-phase state. Combining the average thrust and average lift force acting on the prototype with different phase lags, the swimming attitude control of the robotic fish can be realized by changing the phase lags. Meanwhile, it can also be obtained that the control parameters with better propulsion performance under the synergistic interaction of the pectoral fins and caudal fin of the prototype are: $\Delta \varphi_{14}=180^{{}^{\circ}}$, $\Delta \varphi_{23}=90^{{}^{\circ}}$.

#### 4.5.2. Effects of the Frequency on Propulsion Performance

**Figure 17:**
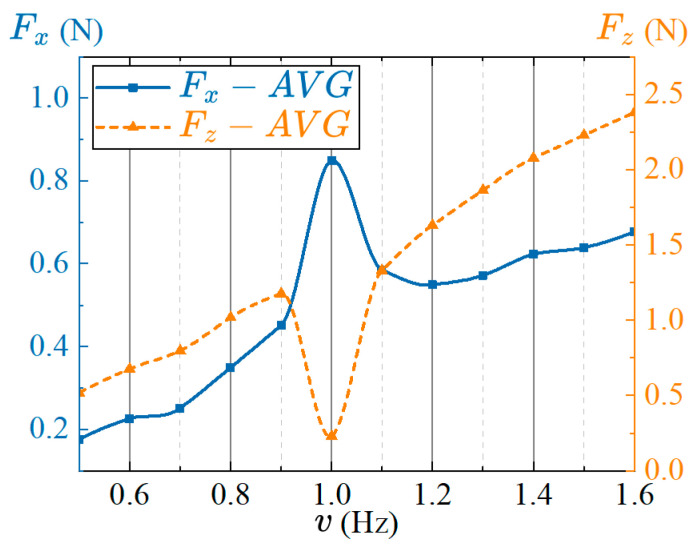
Effects of the frequency on propulsion performance under the synergistical interaction between pectoral fins and caudal fin.

Under the condition of synergistic interaction between pectoral fins and the caudal fin, the phase lags between the actuators is effective when the swing frequency between the CPG units based on the phase oscillator model is the same. Therefore, the frequency values of the oscillator units connected to the network with the same CPG topology must be consistent. As we analyzed in previous sections, the frequency will affect the swing speed of the pectoral fin and caudal fin, then changing the force between the fin surface and the water, thereby affecting the swimming performance of the robotic fish. In order to measure the effects of frequency on the thrust and lift force under the synergistic coupled propulsion of the pectoral fins and tail fin of the robotic fish, the frequency is utilized as the control variable, and the other control parameters are: $R_{1}=R_{4}=40^{{}^{\circ}}$, $R_{2}=R_{5}=30^{{}^{\circ}}$, $R_{3}=15^{{}^{\circ}}$, $R_{6}=20^{{}^{\circ}}$, $X_{i}=0^{{}^{\circ}}(i=1,\cdots ,6)$, $\Delta \varphi_{14}=180^{{}^{\circ}}$, $\Delta \varphi_{23}=90^{{}^{\circ}}$. The frequency ranges from 0.5 Hz to 1.6 Hz with an interval of 0.1 Hz. The average thrust and lift force are measured in several cycles after the stable swing of fins, and the results are shown in Figure 17.

As shown in Figure 17, the average thrust of the robotic fish increases first, then decreases, and then increases as the frequency increases under the condition of the synergistic interaction between the pectoral fins and caudal fin, and the value is the largest at the frequency of 1 Hz. If the average thrust data at 1 Hz are ignored, there is a positive correlation between the average thrust and frequency, that is, an approximately linear increase. The average lift force first increases and then decreases with the increase of frequency, and then increases again, and reaches the minimum value at the frequency of 1 Hz. Similarly, if the average lift force at 1 Hz is ignored, the average lift increases approximately linearly with frequency. To sum up, the optimal frequency of the propulsion performance of the robotic fish under the coupling and synergistic interaction between the pectoral fins and caudal fin is 1 Hz. In addition, the speed adjustment and attitude control of the robot fish can be realized by appropriately changing the swing frequency of the pectoral fins and caudal fin.

#### 4.5.3. Actual Swimming Speed of Robotic Fish at Different Frequencies

**Figure 18:**
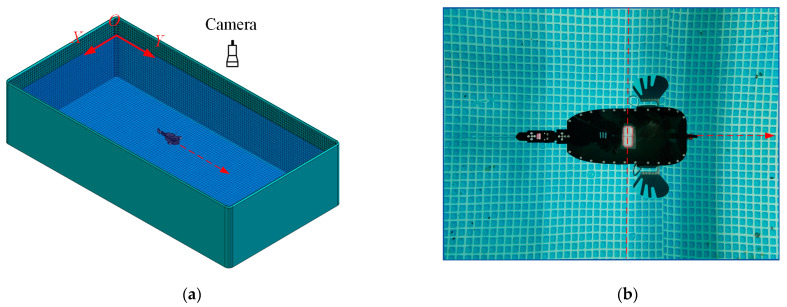
Measuring method of swimming speed. (**a**) Experimental scene of speed test; (**b**) Analysis of swimming speed.

The device and method for testing the speed of the robotic fish in the free-swimming state are shown in Figure 18. The velocity information of the prototype while swimming in the XOY plane is captured at a frequency of 30 frames per second (FPS) (by hanging a high-definition video camera above the tested pool). The captured swimming video was processed by video processing software to measure the time required for the robot fish to swim a specified distance, and then the average swimming speed was obtained. In order to recognize the swimming of the prototype more clearly, a waterproof schematic label of the center of mass was attached to the projection of the center of mass on the back of the prototype, as shown in Figure 18b. In the video processing, each small square grid at the bottom of the pool has a side length of 30 mm, and the swimming speed of the prototype is determined by dividing the grid with a known distance and intercepting the time frame rate of the video when the center-of-mass marking point of the prototype coincides with the vertical line of the grid, thus calculating the time for the prototype to move for a specific length of time.

**Figure 19:**
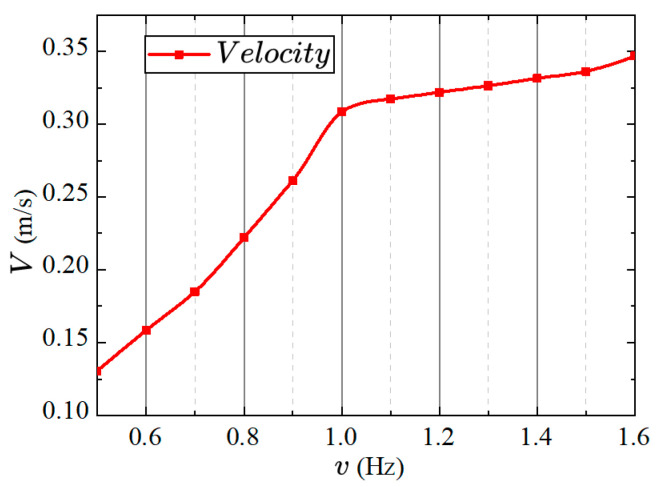
Relationship between forward swimming speed and frequency in robotic fish.

The previous section measured the effects of frequency on average thrust and lift under synergistical interaction between the pectoral and caudal fins. The variation trends of the prototype’s average thrust and lift with respect to frequency were obtained. In this section, in order to better determine the influence of frequency on the swimming performance of the robotic fish, the speed of the fish in the forward swimming state was measured using a video processing method. The resulting variation of swimming speed with frequency is shown in Figure 19.

In Figure 19, the swimming speed of the robotic fish shows a proportional relationship with frequency, indicating that the speed increases with the increase in the oscillation frequency of the pectoral and caudal fins. Additionally, the variation curve exhibits two distinct segments at a frequency of 1 Hz. The slope of the curve before 1 Hz is greater than the slope after 1 Hz. This characteristic suggests that increasing the oscillation frequency significantly improves the swimming performance of the robotic fish at lower frequencies (below 1 Hz). However, at higher frequencies (above 1 Hz), increasing the oscillation frequency only slightly enhances the swimming speed. It is important to note that excessively high frequencies can lead to increased energy consumption, indicating that a high oscillation frequency of the pectoral and caudal fins does not necessarily improve propulsion efficiency. Furthermore, this section differs slightly from the previous section regarding the relationship between thrust and frequency. This discrepancy may be attributed to the influence of water flow disturbances. Since the robotic fish is fixed to the force measurement platform, the interaction between the water flow disturbances and the oscillation frequency of the pectoral and caudal fins becomes more pronounced at a frequency of 1 Hz, resulting in a rapid increase in average thrust at this frequency.

## 5. Conclusions and Future Work

In this paper, we developed and constructed a six-DOF bionic boxfish-like robot. The robot features two-DOF pectoral fins capable of twisting and flapping, as well as a caudal fin consisting of two joints in series, enabling free swimming in underwater environments. Utilizing the designed robotic fish, we implemented a CPG control mechanism based on the phase oscillator model to generate the swimming gait. Additionally, we introduced the time asymmetric flapping characteristic equation to analyze the impact of the asymmetric flapping characteristics of the pectoral fin on propulsion performance. Through hydrodynamic experimental testing, we systematically investigated the effects of the pectoral fins and caudal fin, which are two crucial propulsion joints, on the overall propulsion performance of the robotic fish. We identified CPG control parameters that yielded superior propulsion performance. Our findings indicate that the phase lag between the torsional joints of the pectoral fins on both sides, denoted as $180^{{}^{\circ}}$, and the phase lag between the twisting joint of the pectoral fins and the swing joint of the caudal fin, denoted as $90^{{}^{\circ}}$ or $270^{{}^{\circ}}$, result in better propulsion performance when there is synergistic interaction between the pectoral fins and the caudal fin. Furthermore, we examined the effects of the swing frequency of the pectoral and caudal fins on the propulsion performance of the prototype. Generally, there is a positive correlation between the swing frequency and the average thrust and lift force. However, the best propulsion performance is achieved when the swing frequencies of the pectoral fins and caudal fin are set to 1 Hz, resulting in a large thrust and a small lift force. The analysis method employed in this study holds significant guiding implications for enhancing the swimming performance of robotic fish. Moreover, it provides valuable insights for the motion control of robotic fish driven by multiple fins, including pectoral fins and the caudal fin.

In the future, we plan to enhance the capabilities of our prototype by implementing quantitative control of the depth and heading of the robotic fish. This will be achieved through information feedback from the IMU and depth sensor. Additionally, we will explore the possibility of incorporating speed sensors and vision sensors to optimize the CPG control parameters. These advancements will enable our robotic fish to achieve improved swimming performance and realize additional functionalities through the synergistic interaction of the pectoral and caudal fins. Furthermore, we recognize the importance of studying the effects of various fin surface characteristics, such as fin rays, wing membrane, and aspect ratio, as well as the installation position on the propulsion performance. Additionally, we are interested in investigating the combined effects of the pectoral, dorsal, anal, and caudal fins on swimming performance. These areas of research will contribute to the development of a boxfish-like robot that can be driven synergistically by multiple fins and adapt to diverse working environments. We are excited about the future prospects of our research and look forward to advancing the field of bionic robotics with our innovative developments.

## Figures and Tables

**Table 1:** Parameters of boxfish-like robot fins.

Items	Parameters	Items	Parameters
The twisting angle of pectoral fins	180°	pectoral fin aspect ratio	0.71
The flapping angle of pectoral fins	120°	Length of pectoral fin spread	320 mm
1st oscillation angle of caudal fin	120°	Length of caudal fin spread	535 mm
2nd oscillation angle of caudal fin	150°	Degrees of freedom	6

**Table 2:** Parameters of the CPG phase oscillator model.

Parameters/Units	Abbreviations	Parameters/Units	Abbreviations
Desired amplitude/°	$R_{i}$	Coupling constant	$\omega_{ij}$
Frequency transition coefficient	$k_{vi}$	Swing period/s	$T_{i}$
Instantaneous desired phase lag/°	$\Delta \varphi_{tij}$	Offset angle/°	$x_{i}$
Desired phase lag/°	$\Delta \varphi_{ij}$	Output angle/°	$\theta_{i}$
Time asymmetric coefficient	$\beta_{i}$	Amplitude /°	$r_{i}$
Desired offset angle	$X_{i}$	Frequency/Hz	$v_{i}$
Desired phase lag gain	$c_{ij}$	Offset gain	$b_{i}$
Instantaneous phase/°	$\phi_{i}$	Amplitude gain	$a_{i}$

## Data Availability

Data will be made available on request.

## References

[1] Liu J., Hu H. (2010). Biological inspiration: From carangiform fish to multi-joint robotic fish. J. Bionic Eng..

[2] Kopman V., Porfiri M. (2013). Design, Modeling, and Characterization of a Miniature Robotic Fish for Research and Education in Biomimetics and Bioinspiration. IEEE/ASME Trans. Mechatron..

[3] Jian X., Zou T. (2022). A Review of Locomotion, Control, and Implementation of Robot Fish. J. Intell. Robot. Syst..

[4] Li G., Liu G., Leng D., Fang X., Li G., Wang W. (2023). Underwater Undulating Propulsion Biomimetic Robots: A Review. Biomimetics.

[5] Triantafyllou M.S., Triantafyllou G.S. (1995). An Efficient Swimming Machine. Sci. Am..

[6] Cui Z., Yang Z., Shen L., Jiang H.Z. (2018). Complex modal analysis of the movements of swimming fish propelled by body and/or caudal fin. Wave Motion.

[7] Khan A.H., Ruiz Hussmann K., Powalla D., Hoerner S., Kruusmaa M., Tuhtan J.A. (2022). An open 3D CFD model for the investigation of flow environments experienced by freshwater fish. Ecol. Inform..

[8] Yu J., Tan M., Chen J., Zhang J. (2014). A Survey on CPG-Inspired Control Models and System Implementation. IEEE Trans. Neural Netw. Learn. Syst..

[9] Ahn S.-H., Lee K.-T., Kim H.-J., Wu R., Kim J.-S., Song S.-H. (2012). Smart soft composite: An integrated 3D soft morphing structure using bend-twist coupling of anisotropic materials. Int. J. Precis. Eng. Manuf..

[10] Duraisamy P., Kumar Sidharthan R., Nagarajan Santhanakrishnan M. (2019). Design, Modeling, and Control of Biomimetic Fish Robot: A Review. J. Bionic Eng..

[11] Chen Z., Shatara S., Tan X. (2010). Modeling of Biomimetic Robotic Fish Propelled by An Ionic Polymer–Metal Composite Caudal Fin. IEEE/ASME Trans. Mechatron..

[12] Li Y., Xu Y., Wu Z., Ma L., Guo M., Li Z., Li Y. (2022). A comprehensive review on fish-inspired robots. Int. J. Adv. Robot. Syst..

[13] Zhou Z., Liu J., Yu J. (2022). A Survey of Underwater Multi-Robot Systems. IEEE/CAA J. Autom. Sin..

[14] Fish F.E. (2020). Advantages of aquatic animals as models for bio-inspired drones over present AUV technology. Bioinspir. Biomim..

[15] Breder C.M. (1926). The locomotion of fishes. Zoologica.

[16] Lindsey C.C. (1978). Form, function and locomotory habits in fish. Fish Physiol..

[17] Webb P.W., Rayner J.M.V., Maddock L., Bone Q. (1994). The biology of fish swimming. The Mechanics and Physiology of Animal Swimming.

[18] Raj A., Thakur A. (2016). Fish-inspired robots: Design, sensing, actuation, and autonomy—A review of research. Bioinspir. Biomim..

[19] Costa D., Palmieri G., Palpacelli M.-C., Panebianco L., Scaradozzi D. (2018). Design of a Bio-Inspired Autonomous Underwater Robot. J. Intell. Robot. Syst..

[20] Mignano A.P., Kadapa S., Tangorra J.L., Lauder G.V. (2019). Passing the Wake: Using Multiple Fins to Shape Forces for Swimming. Biomimetics.

[21] Matthews D.G., Lauder G.V. (2021). Fin–fin interactions during locomotion in a simplified biomimetic fish model. .Bioinspir. Biomim..

[22] Zhang T., Wang R., Wang Y., Cheng L., Wang S., Tan M. (2022). Design and Locomotion Control of a Dactylopteridae-Inspired Biomimetic Underwater Vehicle With Hybrid Propulsion. IEEE Trans. Autom. Sci. Eng..

[23] Sharifzadeh M., Jiang Y., Lafmejani A.S., Nichols K., Aukes D. (2021). Maneuverable gait selection for a novel fish-inspired robot using a CMA-ES-assisted workflow. Bioinspir. Biomim..

[24] Drago A., Carryon G., Tangorra J. (2022). Reinforcement learning as a method for tuning CPG controllers for underwater multi-fin propulsion. Proceedings of the 2022 International Conference on Robotics and Automation (ICRA).

[25] Wang W., Dai X., Li L., Gheneti B.H., Ding Y., Yu J., Xie G. (2018). Three-dimensional modeling of a fin-actuated robotic fish with multimodal swimming. IEEE/ASME Trans. Mechatron..

[26] Pollard B., Tallapragada P. (2019). Passive appendages improve the maneuverability of fishlike robots. IEEE/ASME Trans. Mechatron..

[27] Marchese A.D., Onal C.D., Rus D. (2014). Autonomous soft robotic fish capable of escape maneuvers using fluidic elastomer actuators. Soft Robot..

[28] Qiu H., Chen L., Ma X., Bi S., Wang B., Li T. (2023). Analysis of Heading Stability due to Interactions between Pectoral and Caudal Fins in Robotic Boxfish Locomotion. J. Bionic Eng..

[29] Wang W., Xie G. (2016). CPG-based Locomotion Controller Design for a Boxfish-like Robot. Int. J. Adv. Robot. Syst..

[30] Farina S.C., Summers A.P. (2015). Boxed up and ready to go. Nature.

[31] Van Wassenbergh S., van Manen K., Marcroft T.A., Alfaro M.E., Stamhuis E.J. (2015). Boxfish swimming paradox resolved: Forces by the flow of water around the body promote manoeuvrability. J. R. Soc. Interface.

[32] Boute P.G., Van Wassenbergh S., Stamhuis E.J. (2020). Modulating yaw with an unstable rigid body and a course-stabilizing or steering caudal fin in the yellow boxfish (*Ostracion cubicus*). R. Soc. Open Sci..

[33] Blake R. (1979). The mechanics of labriform locomotion: I. Labriform locomotion in the angelfish (*Pterophyllum eimekei*): An analysis of the power stroke. J. Exp. Biol..

[34] Sitorus P.E., Nazaruddin Y.Y., Leksono E., Budiyono A. (2009). Design and Implementation of Paired Pectoral Fins Locomotion of Labriform Fish Applied to a Fish Robot. J. Bionic Eng..

[35] Blake R. (1981). Influence of pectoral fin shape on thrust and drag in labriform locomotion. J. Zool..

[36] Wainwright P.C., Bellwood D.R., Westneat M.W. (2002). Ecomorphology of Locomotion in Labrid Fishes. Environ. Biol. Fishes.

[37] Walker J.A., Westneat M.W. (2002). Performance limits of labriform propulsion and correlates with fin shape and motion. J. Exp. Biol..

[38] Naser F.A., Rashid M.T. (2020). The influence of concave pectoral fin morphology in the performance of labriform swimming robot. Iraqi J. Electr. Electron. Eng..

[39] Delcomyn F. (1980). Neural Basis of Rhythmic Behavior in Animals. Science.

[40] Ijspeert A.J., Crespi A., Ryczko D., Cabelguen J.-M. (2007). From Swimming to Walking with a Salamander Robot Driven by a Spinal Cord Model. Science.

[41] Ijspeert A.J. (2008). Central pattern generators for locomotion control in animals and robots: A review. Neural Netw..

[42] Liu C., Chen Q., Wang D. (2011). CPG-Inspired Workspace Trajectory Generation and Adaptive Locomotion Control for Quadruped Robots. IEEE Trans. Syst. Man Cybern. Part B (Cybern.).

[43] Liu C., Xia L., Zhang C., Chen Q. (2018). Multi-Layered CPG for Adaptive Walking of Quadruped Robots. J. Bionic Eng..

[44] Zhong B., Zhang S., Xu M., Zhou Y., Fang T., Li W. (2018). On a CPG-Based Hexapod Robot: AmphiHex-II With Variable Stiffness Legs. IEEE/ASME Trans. Mechatron..

[45] Ding R., Yu J., Yang Q., Tan M., Zhang J. CPG-based behavior design and implementation for a biomimetic amphibious robot. Proceedings of the 2011 IEEE International Conference on Robotics and Automation.

[46] Wang Z., Gao Q., Zhao H. (2017). CPG-Inspired Locomotion Control for a Snake Robot Basing on Nonlinear Oscillators. J. Intell. Robot. Syst..

[47] Cao Y., Bi S., Cai Y., Wang Y. (2015). Applying central pattern generators to control the robofish with oscillating pectoral fins. Ind. Robot Int. J..

[48] Bal C., Koca G.O., Korkmaz D., Akpolat Z.H., Ay M. (2019). CPG-based autonomous swimming control for multi-tasks of a biomimetic robotic fish. Ocean Eng..

[49] Wu Z., Yu J., Yuan J., Tan M. (2019). Towards a Gliding Robotic Dolphin: Design, Modeling, and Experiments. IEEE/ASME Trans. Mechatron..

[50] Yu J., Wang K., Tan M., Zhang J. (2014). Design and Control of an Embedded Vision Guided Robotic Fish with Multiple Control Surfaces. Sci. World J..

[51] Qiu H., Bi S., Wang B., Cai Y. (2021). Design and Hydrodynamic Analysis of a Robotic Boxfish Using Lift-based and Drag-based Swimming Modes for Propulsion. Proceedings of the 2021 6th International Conference on Robotics and Automation Engineering (ICRAE).

[52] Kato N., Wicaksono B.W., Suzuki Y. (2000). Development of biology-inspired autonomous underwater vehicle “BASS III” with high maneuverability. Proceedings of the 2000 International Symposium on Underwater Technology (Cat. No.00EX418).

[53] Hove J.R., O’Bryan L.M., Gordon M.S., Webb P.W., Weihs D. (2001). Boxfishes (Teleostei: Ostraciidae) as a model system for fishes swimming with many fins: Kinematics. J. Exp. Biol..

